# Use of the O_2_-Thiosemicarbazide System, for the Leaching of: Gold and Copper from WEEE & Silver Contained in Mining Wastes

**DOI:** 10.3390/ma14237329

**Published:** 2021-11-30

**Authors:** Eduardo Cerecedo-Sáenz, Edgar A. Cárdenas-Reyes, Abner H. Rojas-Calva, Ma. Isabel Reyes-Valderrama, Ventura Rodríguez-Lugo, Norman Toro, Edelmira Gálvez, Otilio A. Acevedo-Sandoval, Juan Hernández-Ávila, Eleazar Salinas-Rodríguez

**Affiliations:** 1 Academic Area of Earth Sciences and Materials, Institute of Basic Sciences and Engineering, Autonomous University of the State of Hidalgo, Highway Pachuca-Tulancingo, km. 4.5, Mineral de la Reforma, Pachuca 42184, Mexico; mardenjazz@yahoo.com.mx (E.C.-S.); edgar88cardenas@hotmail.com (E.A.C.-R.); abner_rc@hotmail.es (A.H.R.-C.); profe_5490@uaeh.edu.mx (M.I.R.-V.); ventura.rl65@gmail.com (V.R.-L.); 2 Faculty of Engineering and Architecture, Universidad Arturo Prat, Iquique 1100000, Chile; notoro@unap.cl; 3 Departamento de Ingeniería Metalúrgica y Minas, Universidad Católica del Norte, Antofagasta 1270709, Chile; egalvez@ucn.cl; 4 Academic Area of Chemistry, Institute of Basic Sciences and Engineering, Autonomous University of the State of Hidalgo, Highway Pachuca-Tulancingo, km. 4.5, Mineral de la Reforma, Pachuca 42184, Mexico; acevedo@uaeh.edu.mx

**Keywords:** leaching of gold, leaching of silver, mining wastes, WEEE, O_2_-thiosemicarbazide leaching system

## Abstract

Environmental pollution today is a latent risk for humanity, here the need to recycle waste of all kinds. This work is related to the kinetic study of the leaching of gold and copper contained in waste electrical and electronic equipment (WEEE) and silver contained in mining wastes (MW), using the O_2_-thiosemicarbazide system. The results obtained show that this non-toxic leaching system is adequate for the leaching of said metals. Reaction orders were found ranging from 0 (Cu), 0.93 (Ag), and 2.01 (Au) for the effect of the reagent concentration and maximum recoveries of 77.7% (Cu), 95.8% (Au), and 60% (Ag) were obtained. Likewise, the activation energies found show that the leaching of WEEE is controlled by diffusion (Cu Ea = 9.06 and Au Ea = 18.25 kJ/Kmol), while the leaching of MW (Ea = 45.55 kJ/Kmol) is controlled by the chemical reaction. For the case of stirring rate, it was found a low effect and only particles from WEEE and MW must be suspended in solution to proceed with the leaching. The pH has effect only at values above 8, and finally, for the case of MW, the O_2_ partial pressure has a market effect, going the Ag leaching from 33% at 0.2 atm up to 60% at a 1 atm.

## 1. Introduction

The circular economy is a strategy that seeks to minimize the disposal of waste from the consumption of goods and industrial processes, offering an opportunity to mining and the recycling and reuse of discarded products, to continue growing sustainably, through energy efficiency based on the optimization of resources and secondary or ternary reuse and recycling processes [1,2]. Nowadays, the accelerated rate of industrialization and the demand for better and cheaper goods have led to a higher need for precious and noble metals, while the mineral ores have diminished their quality and contents in nature. The above, join with the increase of severe environmental protection legislation worldwide, has motivated to seek for recycling of wastes to get that wanted metals. In this way, the recovery of precious metals from wastes is an ideal opportunity to get raw materials having additional value, because the involved processes can recover metals without losing their quality, saving energy in comparison with the primary process of recovery, also reducing emissions, decreasing mining activities and the most important, reducing wastes that could be harmful to humankind and environment [3,4,5].

According to the above, there is a great potential in transforming industrial and electronic wastes in materials resources. However, recycling remains at a low level, so there is a needing to work in searching for improvements to the actual technologies applied to recover precious metals from wastes [6,7,8].

However, there are currently a variety of works related to the recovery of precious and base metals contained in industrial and electronic waste (WEEE). These works have focused on recovering these metals through processes that are being improved to avoid contamination, within the remediation that is being carried out. This is how studies have been carried out to recover gold, silver, copper, and other noble metals contained in mining waste and electronic waste, mainly using hydrometallurgical processes [4,7,9,10,11,12].

Traditionally, the recovery of gold and silver was carried out using cyanide. However, this reagent exhibits high toxicity and this has led to the search for less toxic and more selective leaching reagents [1,13,14,15].

Recently, it has been found that the leaching of precious metals using inorganic compounds as leaching reagents, from the THIO group (Thiourea [CS(NH_2)2_], Thiosulfate $\left[S_{2}O_{3}^{2-}\right]$, Thiocyanate [SCN], and Thiosemicarbazide [CH_5_N_3_S]) have some advantages over to the conventional cyaniding process, such as the ease of carrying out the dissolution under acidic conditions using strong oxidizing reagents, likewise, the values can be recovered by means of adsorption or precipitation, and in addition, these kinds of reagents show lower toxicity, reducing thus the environmental problems. Some researchers have achieved thiourea acid leaching of gold and silver of 72% and 86%, respectively [1], although in other cases thiourea was not very successful against cyanide [13,15]. Other researchers saw the possibility of using thiosulfate and ultrasound combined, achieving good silver recovery results [16]. Likewise, ammonium thiosulfate solutions have been used to leach gold evaluating the reagent consumption, improving the leaching of thiosulfate using pretreatment with sodium hydroxide and copper as a catalyst [17,18,19].

Likewise, some kinetic studies have been carried out to evaluate the effect of variables such as reagent concentration, temperature, pH, stirring speed, etc., on mining waste, synthetic solutions, and mineral ores using thiosulfate, finding good results and a promising benefit to apply this type of reactive for the leaching of gold, silver, and some noble metals [11,12,20,21].

In the case of thiosemicarbazide, this is a crystalline basic compound [CH_5_N_3_S], whose melting point ranges between 177 and 179 °C (450 and 452 K) and tends to form salts in the presence of acids [22]. Most thiosemicarbazides dissolve and form metal complexes in alkaline solution and hence their importance in the anionic form in solution [23], occurring a donation to the metal center of one, two, or three electronic pairs and deprotonation due to the loss of hydrogen bound to the hydrazine nitrogen, modifying its behavior to the anionic ligand. Thus, the metal ion can be coordinated by means of a bidentate cis configuration through the sulfur atom and the hydrazine nitrogen atom with the formation of a five-membered chelate ring having great stability, especially with metals of low oxidation state such as the d8 of low spin, like Pd and Pt (II), and the d10 like Cu, Ag, and Au (I), or Hg (II) [24].

This is how this reagent has started to be used for the leaching and recovery of gold from depleted electrolytes using ammonium-thiosemicarbazide, gold adsorption through the use of PVDF membranes functionalized with thiosemicarbazide and poly-thiosemicarbazide membranes [25,26,27,28,29]. Similarly, thiosemicarbazide has been used for the extraction and recovery of silver ions using alumina and 1-((5-nitrofuran-2-yl)methylene) thiosemicarbazide [30].

According to the above, the present work aims to identify the factors that affect, from the kinetic point of view, the leaching of gold and copper contained in WEEE and the silver from the residues of the metallurgical mining industry of Pachuca- Real del Monte, Hidalgo, Mexico. Therefore, it is intended to evaluate the efficiency of the oxygen-thiosemicarbazide system as an alternative, non-toxic and selective leaching for Ag, Ag, and Cu.

## 2. Materials and Methods

### 2.1. Materials

#### 2.1.1. Waste Electrical and Electronic Equipment (WEEE)

**Figure 1:**
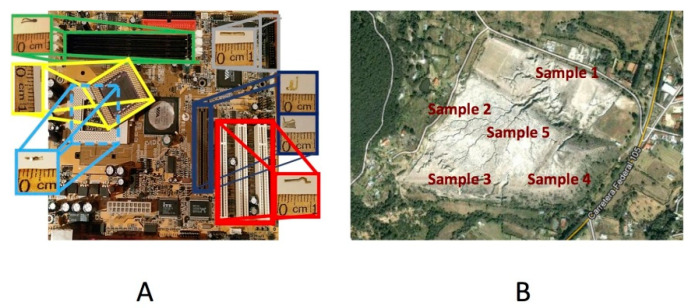
Image of the samples used for the leaching of (**A**) WEEE and (**B**) MW.

For the study of the leaching of gold and copper from WEEE, computer equipment discarded in urban dumps were recovered and used, from where the motherboards were extracted. After this, were collected 22 printed circuit boards for this part of the work, and the metallic compounds supported in such motherboards were dismounted. The so obtained samples (Figure 1A) were later homogenized by a reduction of size cutting them only, for the subsequent chemical characterization to have a starting point on the amount of gold and copper contained in these components, using principally spectrometry of emission by plasma inductively coupled (ICP).

#### 2.1.2. Mining Wastes (MW)

For the leaching study of silver contained in the MW, 5 samples of 50 kg were collected from the “Velasco” dam, located in the State of Hidalgo, Mexico (Figure 1B). The sampling was carried out selectively, taking representative samples from the lower, middle, and upper parts of each point indicated in the image, obtaining a total of five representative samples of the entire waste dump, approximately 50 kg each. Subsequently, the samples were mixed using shovels, homogenized, and by means of consecutive quarters a representative sample of an approximate weight of 1 kg was obtained, for its characterization and leaching study. For this case, the so obtained samples were ground in batch, for 8 min at a working speed of 71.87 rpm with a ball load of 10.23 kg, a pulp load of 2097.77 g, 930 mL of water, and the milling unit (Denver brand) was used with balls mills, from which samples were obtained (all one to 8 min of milling) for subsequent leaching stage.

After the above, samples were again homogenized and quartered to get representative ones which were wholly characterized, using, in this case, the following analytical techniques: X-ray Diffraction (XRD) using a diffractometer INEL model EQUINOX 2000, located at the Autonomous University of the State of Hidalgo (UAEH), Mexico. The diffractograms were obtained at room temperature in an interval of 2θ from 20 to 100°, with Co Kα_1_ radiation (λ = 1.789010 Å). The acquisition time was 25 min, and the evaluation and indexation of the obtained diffraction spectrums were carried out using the MATCH 3 software, which contains the base data COD-Inorg REV 1842382016.07.05 (Powder Diffraction Data Base).

Also was used a scanning electron microscope (SEM) brand JEOL, model JSM-6300 (located at UAEH, Mexico), with a probe current of 10^−2^ to 10^−5^ Amperes (A), and a voltage of 30 keV, to reach 300,000 amplifications. For the morphologic analysis, were utilized the following conditions: acceleration voltage of 30 keV, capacitor current 1 × 10^−9^ A, image mode in secondary electrons (SE), work distance of 15 mm, and 600 µm opening. The determination of the semi-quantitative and punctual chemical composition by energy dispersive spectrometry of X-rays (EDS), was used at an energy of 15 keV with the aid of a spectrophotometer OXFORD.

For the quantitative determination, was used a spectrophotometer (ICP) PERKIN-ELMER, model OPTIMA 3000 XL, which was employed with the plasma of argon as a source for producing ionized atoms, and this equipment is located at the UAEH, Mexico. In a similar manner, by Atomic Absorption Spectrometry (AAS), using a spectrophotometer PERKIN-ELMER model 2380, located at UAEH, Mexico. Finally, the samples were analyzed by X-ray Fluorescence, using a BRUKER handheld XRF spectrometer, model S1 TITAN.

### 2.2. Experimental Procedure

#### 2.2.1. Leaching of WEEE

**Figure 2:**
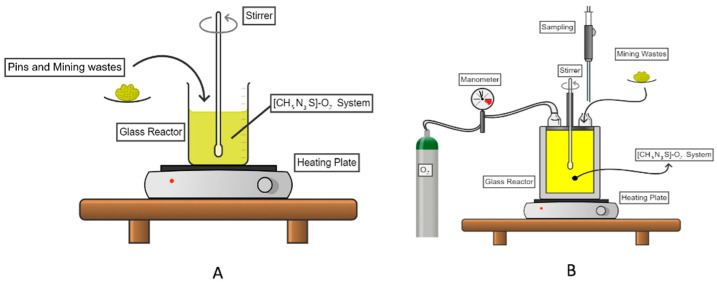
Schematic image of the experimental apparatus used for; (**A**) The leaching of WEEE and MW at open atmospheric conditions, and (**B**) For the study of the effect of O_2_ partial pressure during MW leaching.

The leaching experiments were carried out in a 500 mL conventional flat bottom glass reactor, mounted in a heating plate having magnetic stirring and control of temperature. A pH measurement system was integrated, which consisted of a pH-meter using an electrode of pH suitable for working in extreme acid and alkaline conditions, (pH range from 0 to 14), this experimental system was also used for the leaching of MW and is shown in Figure 2, where Figure 2A is the experimental apparatus used for all the experiments carried out for both materials MW and WEEE, except for the case of the analysis of the effect of O_2_ partial pressure where was used the experimental array showed in Figure 2A. The adjustments of pH during leaching experiments were done by adding a NaOH 2% solution directly to the glass reactor using a graduated burette. The atmosphere was open air inside the reactor (except for the case of MW during the study of the effect of O_2_ partial pressure), and the temperature of the system was controlled by means of a thermocouple attached to the heating rack.

The beginning of the reaction was determined at the exact moment when the sample of WEEE or MW came into contact with the leaching solution. Were taken liquid samples at different time intervals (t). The experimental conditions used in this part of the work are shown in Table 1.

The products obtained in each experiment, both aqueous and solid, were analyzed by spectrometry of emission of plasma inductively coupled (ICP), to determine the copper and gold concentration in solution on each sample taken at preset time intervals.

The quantification of the fraction of leached metals for the kinetics leaching of Cu and Au from WEEE and Ag from MW was calculated using the following expression:

(1) $$X_{Mes}=\frac{Me_{sol}}{Me_{T}}$$

where *X_Mes_* is the fraction of metal in solution (Au, Ag, or Cu), fraction from 0 to 1. *Me_sol_* is the concentration (in ppm) of Au, Ag or Cu in the solution at a determined time (t), and *Me_T_* is the total concentration of Au, Ag, or Cu (in ppm).

The characterization of the obtained products was carried out using different techniques. For the quantitative determination, was used the ICP, considering 6 standard samples, to verify the linearity of the interval of the analysis in parts per million (ppm) of gold (0–30 ppm), and 10 standard samples to verify the linearity of the analysis in ppm for copper (0–700 ppm). From the obtained value in ppm, by the interpolation and knowing the value of the total volume of dissolution and the total amount of sample, were calculated the true ppm of Au and Cu present in each sample.

The characterization of the obtained solids was executed using a Scanning Electron Microscope, and by X-ray diffraction (described both in Section 2.1.2).

#### 2.2.2. Leaching of MW

The experiments for the leaching of silver contained in MW were carried out in a 1000 mL flat-bottom glass reactor, which was mounted in a magnetic stirring heating plate coupled to a mechanical stirrer, which was the same used for the case of the pins in electronic scrap (Figure 2A,B); the pH was continuously measured and adjusted in all experiments with the adding of 0.2 M NaOH solution. For this case, industrial purity oxygen was injected to give a pressure atmosphere inside the reactor in the study of the effect of the O_2_ partial pressure (Figure 2A). Finally, the temperature of the system was controlled by means of a thermocouple attached to the heating grid.

For the determination of the weights for each sample, an OAHUS digital analytical balance was used, Analytical Plus AP210S model with an accuracy of 0.001 mg, located at UAEH, Mexico. The kinetics study of leaching of silver with thiosemicarbazide $\left[CH_{5}N_{3}S\right]$ was carried out under the experimental conditions shown in Table 2.

During the kinetic study, the monitoring of leached Ag at the different preset reaction times from 0 to 240 min, was carried out by Analysis of Ag by Atomic Absorption Spectrometry. Finally, according to the obtained results were determined the activation energy, the effect of thiosemicarbazide concentration, stirring rate, pH, and oxygen partial pressure on leaching rate for determining the kinetics parameters that describe the overall reaction for silver leaching from MW.

## 3. Results

### 3.1. Characterization of the WEEE

A total of 22 motherboards were collected, disassembled and the resulting pines obtained were characterized. The procedure for this characterization consisted in taking 10 g of sample (pines) which were wholly digested using Regia water (3:1; HCl:HNO_3_) and the resulting solution was analyzed by ICP. The obtained concentrations for Au and Cu are shown in Table 3, where can be observed that such concentrations for the case of Au are higher than those found in natural ore mineralization, and for this study, the recovery of these values is of economic and environmental interest.

The obtained concentrations are from representative samples and have an error between them of ±28.18% for copper (due to the high Cu concentration in comparison with Au content and by the use of ICP) and 0.4% for gold, which could be attributed to that the majority of materials employed have a similar elemental distribution.

### 3.2. Characterization of the MW

According to the characterization performed by XRF, ICP and AAS were determined the chemical composition of the mining wastes, which is shown in Table 4.

**Figure 3:**
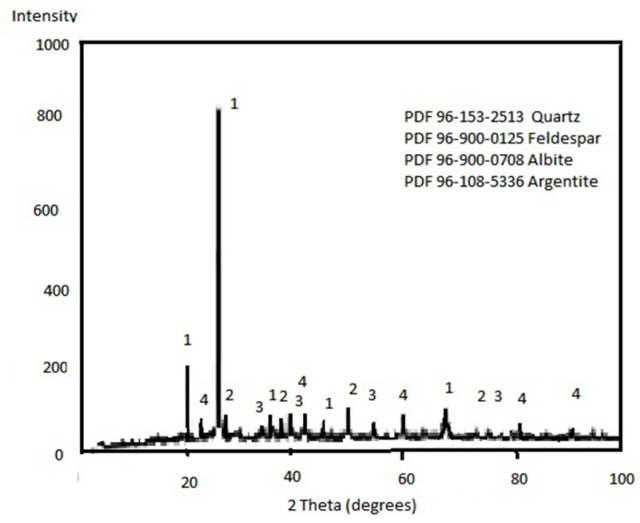
XRD spectrum of the MW from “Velasco” dam.

The principal mineral species identified by XRD were silica, berlinite, orthoclase, feldspar, anorthoclase, calcite, gypsum, hematite, pyrite, sphalerite, galena, and chalcopyrite, which are observed in Figure 3.

**Figure 4:**
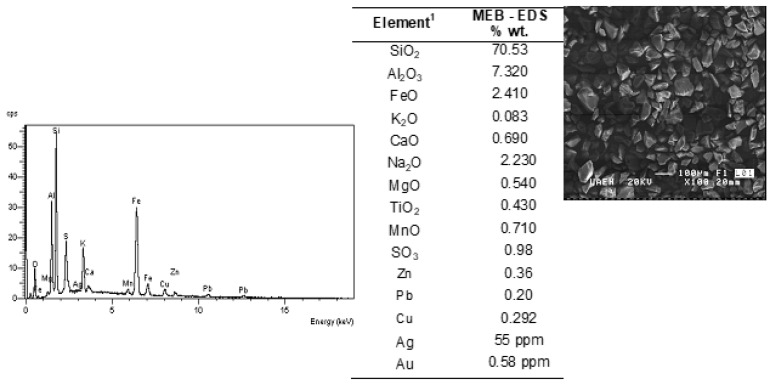
SEM images of MW particles, EDS spectra, and semiquantitative analysis.

On the other hand, Figure 4 shows the general image of particles of the MW, and a particular image of a single particle of material, observing the compact structure of it, and the irregular morphology of the involved particles in the material.

### 3.3. Leaching of Au and Cu from WEEE and Leaching of Ag Contained in the MW

For all leaching experiments carried out for the WEEE and MW, the obtained data were managed according to the decreasing core model for chemical control (Equation (2)) from were obtained the reaction orders (reagent concentration, stirring rate, and pH) and activation energies (effect of temperature) in each case studied [31,32].

(2) [1−(1−XMe)13]=kexp ×t 

where

(3) $$k_{exp}=\frac{V_{M}k_{q}c_{A}^{n}}{r_{0}}\;$$

*X_Me_* is the reacted fraction of Au, Cu, or Ag (from 0 to 1), as corresponding, *V_M_* is the molar volume of material (in m^3^·mol^−1^), *c_A_* is the concentration of leaching reactant (for this case is the thiosemicarbazide, (in mol·m*^−^*^3^), *k_q_* is the kinetic coefficient (in s^−1^), *r*_0_ is the initial radius of the particle in meters (in average), *k_exp_* is the experimental constant in s^−1^, *t* is the time (in seconds) of reaction and *n* is the order of the reaction.

#### 3.3.1. Effect of the Thiosemicarbazide Concentration $\left[{\mathrm{CH}}_{5}N_{3}S\right]$

Firstly, to determine the effect of the leaching reagent on the degree of dissolution of Au and Cu (from WEEE), several experiments at different concentrations of $\left[{\mathrm{CH}}_{5}N_{3}S\right]$ were carried out under the conditions described in Table 1, using, in this case, a constant pH value of 10.5, and as for the case of leaching of silver from MW, the leaching process was executed under the conditions shown in Table 2 for the effect of the tiosemicarbazide concentration.

**Figure 5:**
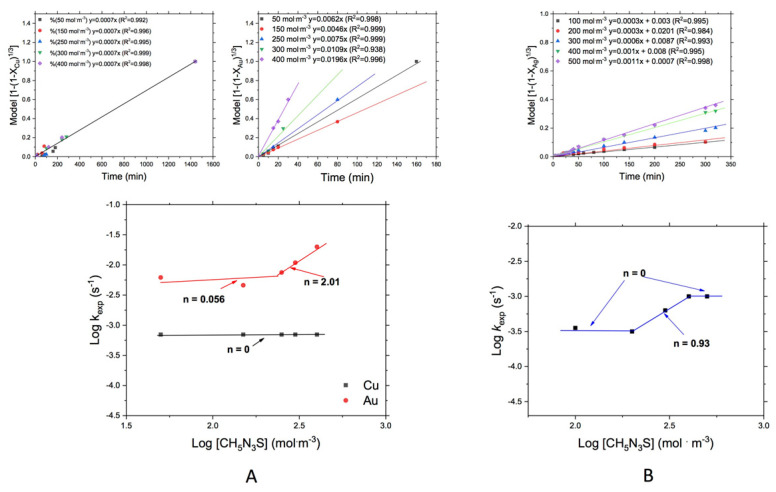
Leaching of (**A**) Au and Cu from WEEE, and (**B**) Ag from MW. Effect of $\left[{\mathrm{CH}}_{5}N_{3}S\right]$.

Figure 5A shows the leached fractions of Cu and Au that were analyzed by the decreasing core model and chemical control (Equation (2)) [31,32]. Straight lines were obtained, and their slopes represented the experimental constants (k_exp_) and can be observed that in the case of Cu there was no apparent effect of thiosemicarbazide concentration on the reaction rate, while for the case of Au can be observed that there is no effect in the range of 50 to 150 mol/m^3^, but passing this range the reaction rate increases while reagent concentration also increases. From these plots were obtained the kinetics values of this variable on the leaching of Au and Cu, where can be observed that for the gold, an order of reaction of *n* = 2.01 was obtained in the range of concentrations from 150 to 400 mol m^−3^, showing a change of order of *n* = 0.056 in the range of concentrations from 50 to 150 mol∙m^−3^, which represents that at low reagent concentration there was no effect of reagent concentration on the leaching rate, but increasing the concentration above 150 mol∙m^−3^ also increase the leaching rates obtained for the leaching of this metal. On the other hand, also is observed that for the case of Cu, there was no effect of reagent concentration on the leaching of this metal contained in the WEEE, practically all k_exp_ was maintained constant.

The effect of $\left[{\mathrm{CH}}_{5}N_{3}S\right]$ concentration on the Ag leaching rate from MW (Figure 5B), also was evaluated with the decreasing core model and chemical control described in Equation (2), showing, in this case, a behavior similar to that shown by Au and in this plot only can appreciate the effect of reactant in the range from 200 to 400 mol∙m^−^^3^ $\left[{\mathrm{CH}}_{5}N_{3}S\right]$. The treatment of the obtained *k_exp_* shows a similar behavior like in the case of Au from WEEE, where an order of reaction of 0 was observed in the range between 100 and 200 mol∙m^−^^3^ $\left[{\mathrm{CH}}_{5}N_{3}S\right]$ and increases to 0.93 in the range between 200 and 400 mol∙m^−^^3^ $\left[{\mathrm{CH}}_{5}N_{3}S\right]$, and finally fell in the range between 400 and 500 mol∙m^−3^ $\left[{\mathrm{CH}}_{5}N_{3}S\right]$ giving again an order of reaction of 0. In the same figure, it is noted that above 400 mol∙m^−3^ and below 200 mol∙m^−3^, there was a change of the order of reaction, reaching a value of 0, which means that at this particular point there was a change in the reaction mechanism, being in the first stage (from 100 to 200), due to the chemical reaction is fast but the diffusion of reactant to particle surface is slow. Above 400 mol∙m^−3^, the excess of reactant could retard the diffusion of products of reaction to inner of solution.

#### 3.3.2. Effect of the Temperature

**Figure 6:**
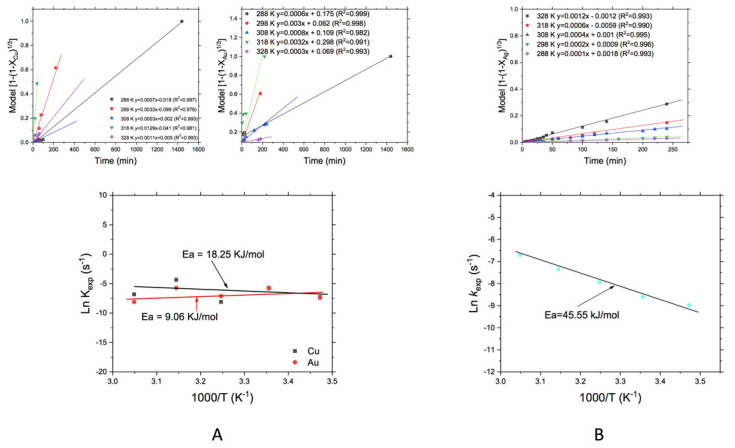
Leaching of; (**A**) Au and Cu from WEEE, and (**B**) Ag from MW: Effect of temperature.

The effect of temperature on the leaching of WEEE and MW is shown in Figure 6. In the upper part of Figure 6A can be observed no significant effect of temperature on reaction rate (*k_exp_*). Meanwhile, the bottom part of the plot shows the activation energies obtained from the treatment of *k_exp_* data according to the Arrhenius equation [31,32] where the slope obtained (m = −(E_a_/R) of the linear curve represented the activation energy (E_a_) divided by the negative value of the universal gas constant (Figure 6A,B), for the leaching of Cu and Au contained in the WEEE. Figure 6B shows the *k_exp_* obtained by the treatment of data with the decreasing core model and chemical control (Equation (2)) and in the lower part of the plot is shown the activation energy obtained after the leaching of Ag from the MW.

The activation energies (in the range from 288 to 328 K) obtained for Cu and Au from WEEE are shown in Figure 6A, where can be observed for the case of Cu an E_a_ = 9.06 kJ/mol, which is indicative of a process controlled by diffusion. Practically appears that temperature, in this case, had no important effect on the leaching rate, but was observed that above 318 K there was a decomposition of the leaching reagent which was observed according to a decreasing of the leaching rate (*k_exp_*). On the other hand, the E_a_ obtained for the leaching of Au from WEEE was 18.25 kJ/mol that also is indicative of a process controlled by diffusion, and like was observed for the leaching of Cu, the behavior was quite similar. For the case of the MW, the activation energy obtained for the leaching of silver was 45.55 kJ∙mol^−1^, which fell in the range of 288 to 328 K (15 to 55 °C) and describes that chemical reaction is the controlling stage in the leaching of silver contained in MW, in the O_2_–thiosemicarbazide system.

#### 3.3.3. Effect of the Stirring Rate

**Figure 7:**
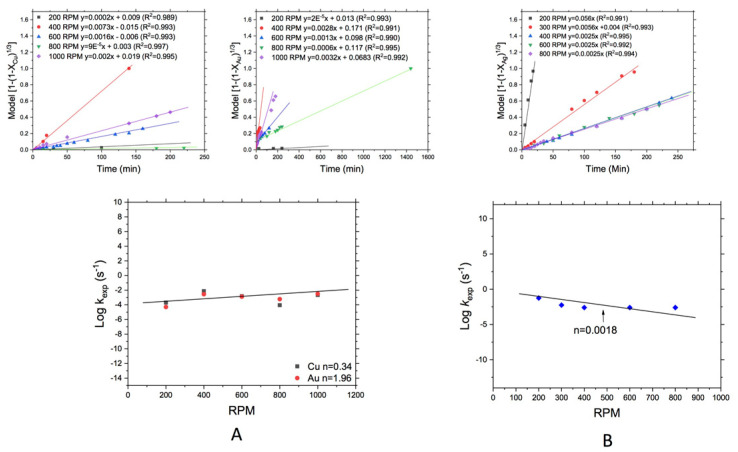
Leaching of (**A**) Au and Cu from WEEE, and (**B**) Ag from MW: Effect of stirring rate.

For the case of the effect of the stirring rate on the leaching of Cu and Au from WEEE and Ag from MW, the results are shown in Figure 7. Figure 7A shows in the upper part, the obtained k*_exp_*, where can be observed that the increase of stirring rate improves reaction rate in both leached metals (Cu and Au), and this is logical due that the obtained activation energies correspond to a diffusion control and the stirring can break the liquid-solid film improving the leaching. The behavior of this variable on the leaching of Cu and Au, where can be observed that apparently there is no significant effect of the stirring rate in the leaching of both metals, although for the case of Cu the order of reaction was *n* = 0.34 while for Au the order of reaction was of *n* = 1.96, perhaps because in the case of gold, activation energy was lower than for the case of Cu where the mass transfer is slower and the need of more stirring rate is more marked. Also was observed that at 400 rpm in both metals the leaching rate was adequate and that was because at that stirring rate particles are well dispersed in solution breaking so the liquid-solid film promoting so, the pass of reagent to the surface of particles. On the other hand, the effect of stirring rate for the leaching of Ag from MW is shown in Figure 7B, where can be observed practically no effect of this variable on the Ag leaching (*n* = 0.0018), which could be due to the chemical control observed during the process of leaching and determined by the activation energy found, only in this case particles needed to be well dispersed in solution.

#### 3.3.4. Effect of the pH

**Figure 8:**
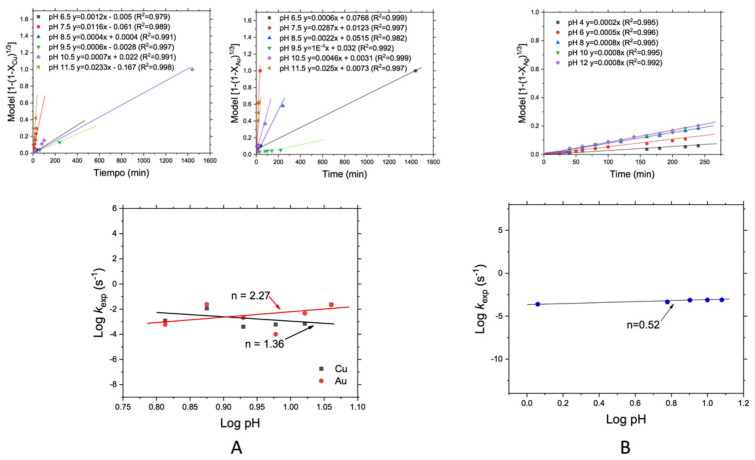
Leaching of (**A**) Au and Cu (from WEEE) and (**B**) Ag (from MW). Effect of the pH.

Figure 8A shows the effect of pH during leaching of Cu and Au from WEEE and Figure 8B shows the same effect for the leaching of Ag from MW.

In Figure 8A, an order of reaction for the effect of pH on Cu leaching of *n* = 1.36 can be observed, while for the case of Au the order of reaction reached was *n* = 2.27 which is indicative that for both cases, the pH value affects alkaline conditions improving the leaching process, in both cases, it was observed that leaching rate increased at higher pH values.

Finally, the effect of the pH for the Ag leaching is represented in Figure 8B where can be seen that this variable has no significant effect on Ag leaching rate in the range from 4 to 12, confirming again that oxygen concentration strongly promotes the Ag leaching from MW, due to the chemical control found for this case.

#### 3.3.5. Leaching of Ag from MW: Effect of the O_2_ Partial Pressure

**Figure 9:**
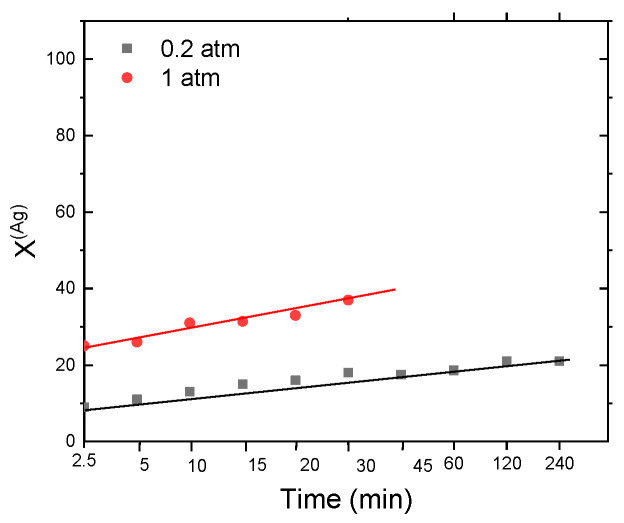
Leaching of Ag from MW: Effect of O_2_ partial pressure.

For the case of leaching of Ag from MW, the effect of O_2_ partial pressure was evaluated, which is shown in Figure 9, where can be observed a strong dependence of this variable on the silver leaching rate, increasing the recovery of silver from 33% in a 0.2 atm O_2_ partial pressure up to 60% in a 1 atm O_2_ partial pressure, which confirms that increasing oxygen in system leaching reaction also is improved.

**Figure 10:**
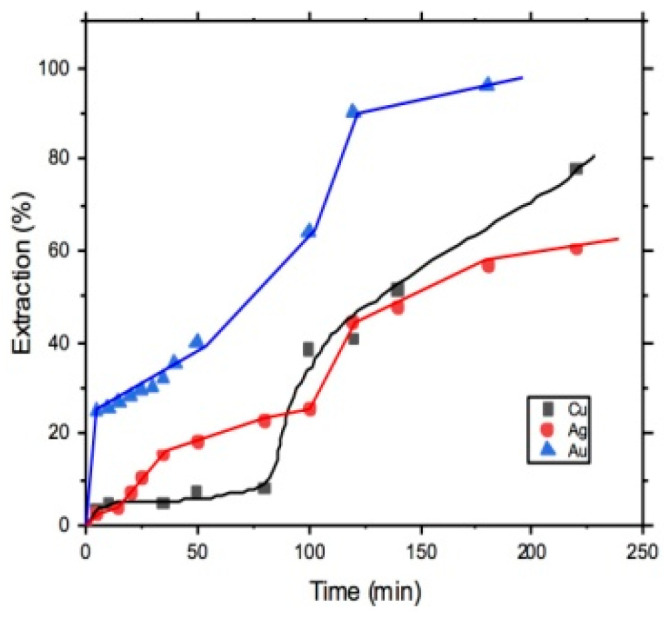
Maximum recoveries of Au, Cu, and Ag were obtained with the best leaching conditions.

Finally, Figure 10 shows the maximum extraction reached for Au, Au, and Cu, according to the best leaching condition found during the experimental study. For the case of Cu and Au, the experimental condition were 400 mol∙m^−3^ $\left[{\mathrm{CH}}_{5}N_{3}S\right]$, 318 K, 400 rpm of stirring rate, and pH of 7.5, obtaining 77.7% of Cu extraction and 95.8% of Au extraction. For the case of Ag leaching, the best condition were 500 mol∙m^−3^ $\left[{\mathrm{CH}}_{5}N_{3}S\right]$, 318 K, 300 rpm of stirring rate, 1 atm of O_2_partial pressure, and pH of 8, obtaining 60% of Ag extraction.

#### 3.3.6. Solid by-Products from WEEE and Kinetic Equations for Leaching of WEEE and MW

**Figure 11:**
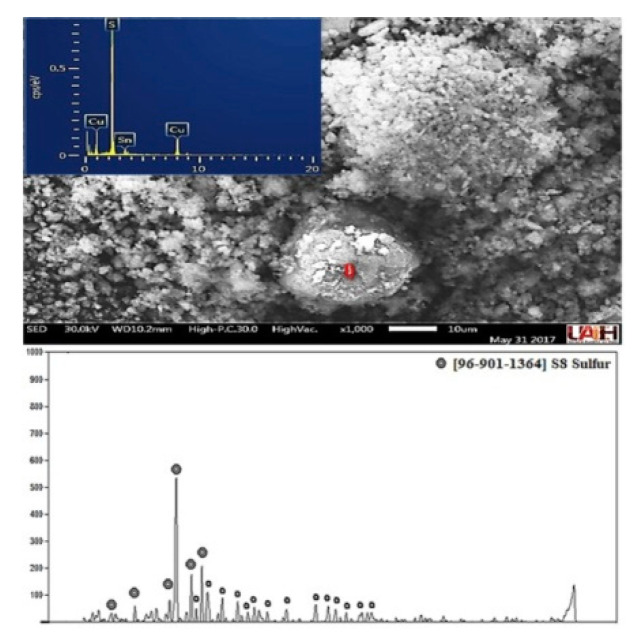
Solid residue image obtained after leaching of WEEE.

In all experiments done, at the end of the leaching process, a solid reside of doughy consistency was obtained, which once characterized by SEM–EDS and XRD, was found to be the sulfur majority with 62.88 Wt %, 26.67 Wt % *Cu* and 10.45 Wt % Sn (EDS analysis, semi-quantitative and punctual analysis) as is shown in Figure 11. The above could be attributed to the formation of links between *S* and *Cu*, and this reaction can be only considered in the experimental conditions studied here, where the formation of such by-products correspond to sulfurs (*Cu_2_S*) and forming also intermediate products like *CuS* with the ions into the solution, according to the following proposed reaction:

(4) Cu2S+2H++12O2 →CuS+Cu2++H2O

According to the results shown above, the kinetics expression determined in the $O_{2}-[{\mathrm{CH}}_{5}N_{3}S]$ media for the leaching of Cu (from WEEE) in the range of reagent concentrations from 50 to 400 mol $\left[{\mathrm{CH}}_{5}N_{3}S\right]$∙m^−3^ for Cu, represented as follows:

(5) r0VM[1−(1−X)13]=1.34 × 103 exp(−1825/RT) [CH5N3S]0 · t

For the leaching of Au (from WEEE) in the range of reagent concentrations from 150 to 400 mol $\left[{\mathrm{CH}}_{5}N_{3}S\right]$∙m^−3^, the kinetics expression is represented as follows:

(6) r0VM[1−(1−X)13]=35.23 exp(−9060/RT) [CH5N3S]2.01 · t

On the other hand, for the leaching of Ag contained in the MW and according to the results found in this study, the kinetics expression determined in the $O_{2}-[{\mathrm{CH}}_{5}N_{3}S]$ media, can be represented as follows for the range of reagent concentrations from 200 to 400 mol $\left[{\mathrm{CH}}_{5}N_{3}S\right]$∙m^−3^:

(7) r0VM[1−(1−X)13]=1.84 × 104 exp(−45550/RT) [CH5N3S]0.93 · t

where *V_M_* = 265.0 × 10^−6^ m^3^∙mol^−1^ (for MW particles), *R* = 8.314 J∙mol^−1^ K^−1^, *r*_0_ in m, T in degrees Kelvin, [CH_5_N_3_S] is in mol∙m^−3^, and *t* is in seconds.

Finally, the overall reaction carried out during the leaching process could be defined by a complexation reaction between the *d* orbital of metal and the pair of free electrons from S present in the structure of the complex binder of thiosemicarbazide with *Ag (I)*, and *Au(I)*, respectively. This is represented with the following formulas:

(8) $$Ag+CH_{5}N_{3}\;\to \left[Ag\left(CH_{5}N_{3}S\right)\right]$$

(9) $$Au+CH_{5}N_{3}\;\to \left[Au\left(CH_{5}N_{3}S\right)\right]$$

## 4. Discussion

According to the results found in this work, both WEEE and MW have interesting Au, Cu, and Ag values to be processed by dynamic leaching using the O_2_-thiosemicarbazide system. These values are consistent with those reported in different works [1,5,7,11,12,21].

For the case of leaching of WEEE and MW, evaluating the concentration of thiosemicarbazide, it was found an oscillating effect for Au and Ag (Figure 5) that could be attributed to the fact that thiosemicarbazide has a thione group (C=S) inside its structure and another free group (–NH_2_), that according to the Pearson´s principle correspond to a chelating behavior, and such chelating effect causes bulky divalent ions to be coordinated either through the sulfur atom (thione) with a soft character or via the nitrogen atom (amine) which has an intermediate character; that is between hard and soft. According to this, the existing bonds can be broken, forming different complexes assigned to the variety of metallic species in the pins, forming a cycle of absorption and desorption of metallic species. The phenomenon of a reaction order change (Au and Ag) also could be due to that at low concentrations of thiosemicarbazide, the gold and silver leaching rate depends essentially on the complexing itself and the partial pressure of oxygen; however, as can be observed in the above Figure 5, from a certain concentration of thiosemicarbazide, the leaching rate remains stable and it is not affected by the concentration of the complexing, passing the leaching rate to depend solely on the dissolved oxygen concentration in the system, like was determined for another authors that used thiosulfate for leaching of Au and Ag in the presence of Cu [12,17,18,20,21,33].

On the other hand, the obtained activation energies for Cu (18.25 kJ/mol) and Au (9.06 kJ/mol) are similar to those found in other works using thiosulfate, where the leaching kinetics process could be controlled by mass transfer of oxygen at the solid-liquid interface [12], also in this work was noted that the presence of Cu and excess of O_2_ promoted that reaction rate increases with respect to the process without Cu, however, in this case, the Cu also was complexed by the thiosemicarbazide and for that reason, here Cu was not acting completely as the oxidant [18,20]. For the case of Ag from MW, the activation energy found (45.55 kJ/mol), shows that the leaching kinetics, in this case, is controlled by the chemical reaction, which is comparative with that works related to the use of thiosulfate on ore minerals where some species could act like oxidants, such as copper or cobalt contained in the ore itself or with the addition of copper or cobalt; improving the mass transfers of reaction product through the solid-liquid interface and retarding so the chemical reaction [21,33,34]. Other authors have pointed that excess temperature (above 323 K) decreased the amount of gold (or silver) dissolved, because this could promote that reactant species decreased their activity due to a decomposition reached at higher temperatures, which could be occurring here, so like in the case of the thiosulfate, the thiosemicarbazide can also be decomposed forming colloidal sulfur, retarding the reaction through surface passivation [1], as well as passivation due to cupric sulfide, formed by the thermal reaction between Cu(II) ions and thiosulfate, since at 60 °C the kinetics of cupric sulfide formation is very fast, preventing the dissolution of gold [35].

For the effect of stirring rate and pH, it was found principally no significant effect of the first parameter on leaching of Cu from WEEE and Ag from MW. In the first case due possibly to the fact that Cu also acts as an oxidant, and only the particles must be suspended to proceed with the reaction, while in the case of Ag because the mass transfer is fast (due to chemical control), there was no effect of stirring rate on the leaching rate of the process. On the other hand, on the leaching of Au, the stirring rate has an order of 1.96 indicating that at elevated values of this variable the leaching rate increases due to the breaking of solid-liquid interfaces, such as was noted in other works [12,17,20].

For the pH effect, a marked difference can be observed for the other values of pH evaluated, which can be due to the stability of the leaching reagent in such pH value (8.5). These values are consistent with those found by López de Alva [36] and Mudasir Ahmad et al., [37], in relation to the recovery of Cu. However, in this work, was obtained a good amount of gold dissolution in different processing conditions, because it is present in a heterogeneous mixture of metals and alloys, limiting so the today existing extraction methods. In this case, the excellent absorption can be easily explained according to the sulfur´s Pourbaix diagram (Eh-pH) at 25 °C [38], where can be observed that under the oxidizing conditions at such pH, the formation of water-soluble metals sulfates is facilitated by the presence of metal cations. At low pH, the amount of H^+^ ions is greater, so that many are lodged in the active sites of the leaching reagent, limiting the absorption of metal ions as the pH increases, and the disposition of these sites increases.

In the same way, the effect of O_2_ partial pressure on Ag leaching from MW was quite similar to that reported in other works where the O_2_-thiosulfate system was used on mining tailing, silver powder, and silver plate [12,20,33].

Finally, the obtained results found in this work lead to the possibility of using the O_2_-thiosemicarbazide system directly in leaching processes to dissolve Au, Ag, and Cu from wastes (electronic and or industrial), in a similar way like other authors have used different compounds and membranes formed by ammonia-thiosemicarbazide, and different types of functionalized membranes with this reagent to recover nanoparticles of Au and Ag [25,26,27,28,29].

## 5. Conclusions

This work showed the use of the O_2_-thiosemicarbazide system in the leaching of Cu and Au from WEEE and Ag from MW, evaluating the kinetics behavior for both processes. Reaction orders were found ranging from *n* = 0 for Cu leaching (from 50 to 400 mol∙m^−3^ thiosemicarbazide concentration), *n* = 0.93 for Ag leaching (from 200 to 400 mol∙m^−3^ thiosemicarbazide concentration), and *n* = 2.01 for Au leaching (from 150 to 400 mol∙m^−3^ thiosemicarbazide concentration), for the case of the effect of the reagent concentration maximum recoveries of 77.7% (Cu), 95.8% (Au) and of 60% (Ag), were obtained. Likewise, the activation energies found that the leaching of WEEE is controlled by diffusion (Cu; Ea = 9.06 and Au; Ea = 18.25 kJ/mol), while the leaching of Ag (Ea = 45.55 kJ/mol) from MW is controlled by the chemical reaction. The effect of stirring rate was low and only both particles of WEEE and MW must be adequately suspended in solution to proceed leaching process, however, in the case of gold, high stirring rate values were determinant to break solid-liquid interface promoting the increase of reaction rate, due to mass transfer is slower than the other cases because this controls the overall reaction. Finally, the pH has effect only at values above 8, and for the case of MW, the effect of O_2_ partial pressure has a market effect, going the Ag leaching from 33% at 0.2 atm up to 60% at a 1 atm.

## Figures and Tables

**Table 1:** Experimental conditions were used for the study of leaching of WEEE.

Parameters	Experimental Conditions
$\left[{\mathrm{CH}}_{5}N_{3}S\right]$(mol∙m^−3^)	50; 150; 250; 300 and 400
pH	6.5; 7.5; 8.5; 9.5; 10.5 and 11.5
Sample weight (g)	10
Temperature (K)	288, 298, 308, 318, and 328 K
Stirring system	Mechanical
Pressure	Atmospheric
Stirring rate (RPM)	200, 400, 600, 800, and 1000

**Table 2:** Experimental conditions used for the study of leaching of MW.

Parameters	Experimental Conditions
$\left[{\mathrm{CH}}_{5}N_{3}S\right]$ (mol∙m^−3^)	100; 200; 300; 400 and 500
pH	4; 6; 8; 10 and 12
Sample weight (g)	40
Temperature (K)	288, 298, 308, 318, and 328
Stirring system	Mechanical
Pressure	Atmospheric and 0.2 atm
Stirring rate (RPM)	200, 300, 400, 600 and 800

**Table 3:** Content of gold and copper in the WEEE.

Sample	Element	Concentration (mg/kg)
1	Cu	2211.8027
	Au	23.1853
2	Cu	2275.9366
	Au	23.2759

**Table 4:** Chemical composition of the MW used for leaching of Ag.

Element ^1^	Wt. (%) AAS	Wt (%) XRF
SiO_2_	70.43	73.30
Al_2_O_3_	7.32	6.50
Fe_2_O_3_	2.80	2.80
* SO_3_	1.14	0.94
K_2_O	0.80	0.80
CaO	0.69	0.69
Na_2_O	2.32	0.08
TiO_2_	0.53	0.30
MgO	0.54	0.55
MnO	0.73	0.73
P_2_O_5_	0.12	0.12
Ag	51 g/ton	56 g/ton

^1^ Elements as oxides * Determined by ICP.

## Data Availability

Data is contained within the article.
